# Clinics

**Published:** 1870-01

**Authors:** 


### CLINICS.

### HOSPITAL NOTES AND GLEANINGS.

Reports of Surgical Practice in London Hospitals.—We went to see the operations at University College Hospital, October 27th last, and were glad to find the list unusually attractive, including the ligature of the external iliac artery, by Mr. Erichsen, and four cases of lithotrity, by Sir Henry Thompson.

The first patient brought into the theatre was a cabman, aged 37, who had first noticed a large femoral aneurism in the right groin four weeks previously, and in whom Mr. Erichsen proceeded to tie the external iliac artery. Chloroform was administered by the Resident Medical Officer, with Clover's apparatus, which is now always used at this hospital. As soon as the man was fairly narcotized, Mr. Erichsen commenced, making the incision rather higher than usual, dividing the external oblique muscle without a director, but using this guard when making the deeper incisions. The peritoneum came distinctly into view, with the gut shining through it. No bleeding requiring interference occurred; the artery was speedily reached, and an ordinary stout whipcord ligature put round it. In the course of his subsequent explanatory remarks, Mr. Erichsen said, that the incision had been made higher than usual—so high, indeed, that he could have easily reached the common iliac vessel, had that been desired—in order to get as far above the aneurism as possible, since this extended above Poupart's ligament. He pointed out the various steps of the operation, and said, that he would have employed the carbolized catgut ligature, as used by Professor Lister, but that on trying some of it before coming into the theatre, he had not felt satisfied of its strength; and, as the thread must be placed at the bottom of a tolerably deep wound, he thought it, on the whole, safer to trust to the usual whipcord. He mentioned that there was another aneurism in the hospital, which might yet come before them in that place; but, at present, digital compression was being practised, the aneurism being in the popliteal space, and he considered it right to give both compression and flexion a fair trial, before subjecting the patient, a man aged only 23, to the risk of the graver alternative.

The next case was one of a large, firm nasal polypus, completely blocking the orifice of the right nostril of a woman. Mr. Erichsen slit up the nostril along its junction with the cheek, and cut through the root of the growth with scissors, and afterwards closing the wound with wire sutures and collodion. We carried away a portion of the polypus for microscopic examination, as it did not present the more usual gelatinous appearance of these growths. We found the bulk of it to consist of spindle cells, intermingled with which were some large cells, of the myeloid kind, so that the structure resembled rather that of an epulis, than of an ordinary nasal polypus; and it probably sprang from the periosteum, covering the nasal process of the superior maxilla.

Sir Henry Thompson's four cases of lithotrity now put in their appearance in quick succession, and we were amused with the regularity and uniformity attending the introduction of these patients. The old men came down one after another, each bearing his pillow, on which his buttocks were to be supported, and carrying in his hand a pill-box, containing the results of the former crushings. As each patient entered the theatre, he was greeted by the surgeon with the question: "How often have you been crushed? followed by "Have you your stone with you?" The contents of the pill-box were then displayed, the nature of the calculus being explained to the students, whilst the man climbed on to the table and adjusted his pillow. Then the lithotrite was introduced, the stone instantly caught and crushed two or three times, and the loaded instrument gently withdrawn, being quickly whipped out of the orifice; the only step in the process producing any demonstration on the part of the patient. The extracted fragments were then turned out on to a bit of blotting-paper and added to the pill-box collection, and the patient carried back to bed. Three of the stones were of uric acid, occurring in men aged 65, 57, and 65 respectively, and the fourth was of urate of soda, in a man aged 69, who had been crushed already three times. The others had been before operated upon once, twice, and thrice respectively. In one instance, where an enlarged prostate complicated matters somewhat, the man had suffered from slight febrile disturbance between the crushings.

The last case was one of disease of the os calcis, in which Mr. Erichsen, having cut down upon the bone by a T-shaped incision, gouged out the carious portion, and smoothed down the edges of the cavity with an osteotrite—a practice which, he stated, he invariably adopted when it could be done safely; as, by this means, an exceedingly useful foot might be secured, the disease being prevented from extending to the joint.

Going round the surgical wards of St. Mary's Hospital, with Mr. Haynes Walton's class, one day last week, we were specially struck with the excellent results of the system of continued irrigation, as adopted by that surgeon. One good example of the beneficial effects of this plan of treatment we have lately recorded in these columns. At the time of our report, the man was making good progress towards recovery. He has since left the hospital, with a perfectly strong, albeit somewhat curved, leg. Our readers may remember that the case was one of severe compound and comminuted fracture of the tibia, in which a large bit of the bone was broken off, leaving only a thin plate of the posterior surface of the bone undetached, at the bottom of a large wound. Although this wound took on an actively sloughing character, and the patient's general health became seriously impaired, from the date of the commencement of tepid irrigation, not only was all, or nearly all, pain removed, but the reparative process made such good way that the whole of the new bone was formed with the discharge of only a thin scale, like a large thumb-nail.

There is, at this moment, in one of the male surgical wards, another good example of this treatment, in the form of a very large ulcer nearly surrounding a man's leg. The surface is now covered with bright, healthy granulations, and an inch of sound cicatrical skin surrounds the sore; but at the date of admission, the ulcer was very much larger, the surface exceedingly foul, and the edges spreading rapidly. The phagedæna was checked, and the healthy action set in immediately on the irrigation being applied.

We hope to present more of these cases to the notice of our readers at a future time.

The plan of putting up fractures of the thigh, suggested long since by Mr. Walton, and exclusively used by him in the wards, seems to produce such good results that we may mention here the chief points of the practice, although it has no longer any novelty to recommend it. The main defect in the ordinary long, or "Liston's," splint is, that extension being made from the tarsus, this part of the foot is apt to be elongated, and an inconvenient deformity is thereby induced. In order to prevent this, Mr. Walton directs, that instead of the usual two equal notches in the bottom of the splint, the upper notch (in the position of the applied splint) shall be only one inch long, the lower one being from seven to eight inches. The splint should be long enough to reach well up into the axilla, from an inch below the sole of the foot, and carefully padded. The foot and lower third of the leg are then thickly padded with cotton-wool, so as to prevent any possible pressure on heel, external malleolus, or tendo-Achillis. The splint is now to be applied to the outer side of the limb, and a turn of bandage passed round the metatarsus and through the shorter notch, so as to fix the foot quite squarely in position. The bandage is then carried up over the ankle, and a turn or two round the leg, above the malleoli and through the larger notch, fixes the splint firmly to the leg, after which the bandage may be taken in the usual manner up to the knee. The perineal band is next adjusted, and counter-extension made, the splint being further fastened by a broad bandage passed round the pelvis, between the great trochanter and the crest of the ilium, and the head of the splint kept in a comfortable position by a bandage round the chest, which may be altered by the patient to suit his own comfort. The thigh has thus no bandage covering it, although the whole apparatus is so firmly fixed that the patient may be readily rolled over on to his sound side, without disturbing the limb. The perineal band may be removed by the end of a week, the muscles being then quite quiet. During the period of confinement to bed, the heel is never allowed to rest for long together in one position, pillows being shifted daily under the leg, so as to prevent any chance of the formation of that most tedious sore to cure, which results from a slough on the heel.

We saw a curious case, also under the care of Mr. Haynes Walton, of a man, aged 35, who, three years and a-half before, had sustained a severe fall, in which he had lost one of his front teeth—the right upper latteral incisor. A few weeks later, after much pain and swelling in the upper jaw, an abscess formed, and discharged through a small opening in the cheek, as well as through the alveolus of the lost tooth. The case was supposed to be one of caries of the maxilla, and many naval surgeons treated the man under this belief, but without alleviating the symptoms. When he came under Mr. Walton's care, there was considerable swelling of the side of the face, much pain, and constant profuse discharge from a sinus in the right cheek, half an inch outside the nostril. The discharge from the alveolus had stopped, and the bottom healed over. On probing the sinus in the cheek, Mr. Walton was convinced that he detected the smooth, hard surface of tooth enamel, and he accordingly enlarged the opening and extracted, with forceps, a perfect incisor tooth, lying loose in the antrum. When we saw the man, eleven days after the operation, the wound was all but healed, and the pain and swelling quite disappeared.—*Medical Times and Gazette*, November 6th, 1869.

---

Excision of Head of Femur, with Great Trochanter, for Hip-joint Disease; subsequent Perforation of the Floor of the Acetabulum, from Extensive Caries; Death.—A point of great interest in the following case, which was treated in the "Dreadnought" Hospital Ship, is the information it affords regarding the influence of resection of the hip, on the progress of carious disease in the acetabulum. It has been recently suggested that caries of the floor of this cavity need not be considered as a contra-indication of the operation, or even as a likely cause of its failure, since the removal of the diseased head of the femur from its socket and a large external wound would favor the elimination of the carious bone, by natural means, and ultimate cure. The clinical history of Mr. Rook's patient, however, proves the necessity of hesitation and great caution in applying these views in practice. At the time of the operation, the chief seat of the disease was the upper end of the femur, including the neck and the two trochanters; this was detached by the saw and removed, exposing the floor of the acetabulum, which, though stripped of its articular cartilage, was composed of hard and apparently sound bone, and presented but slight traces of active disease. The case, at first, promised favorably, but, at the end of the fourth week the progress towards recovery was arrested, and profuse suppuration, acute pain in the hip, and, finally, symptoms indicative of intra-pelvic effusion, resulted in the death of the patient, fourteen weeks after the excision. At the autopsy, the upper extremity of the femur was found capped with organized lymph and in a very satisfactory condition. The acetabulum, on the other hand, was extensively affected, its floor perforated in several places, and the surrounding bone converted into an uneven, soft, and carious mass—the result of disease, the activity and speed of which had increased to a very great extent after the removal of the head of the femur.

Joseph F., aged 18 years, a thin and excitable lad, was admitted into the Seamen's Hospital, on October 21st, 1868.

*Previous History.*—Commenced a seafaring life as a cook on board a coasting vessel, in the spring of 1864. Had very good health up to the month of November, of the same year, when, after prolonged exposure to wet and cold, he complained of general uneasiness, and subsequently of stiffness and severe pain in the right hip. The local symptoms increased in severity, and, in spite of treatment and rest for nine months, in the Swansea Infirmary, rendered the lad perfectly helpless. He was then sent to London, and resided with a relation up to the time of his admission into the "Dreadnought." When first seen by Mr. Rooke, the patient presented well-marked symptoms of morbus coxarius. There was no abscess. He was kept at perfect rest in bed, and treated by the administration of tonics and the occasional application of the actual cautery. At the end of the year, he had recovered so far, that he was allowed to get up. The joint, at this time, was free from pain. A heavy fall in the ward, in February, 1869, was followed by acute pain in the hip, painful muscular spasms, and severe general reaction. Abscesses formed rapidly at the upper part of the thigh, from which there was a profuse discharge of thick, greenish pus, which could not be arrested. In consequence of the increasing debility and emaciation of the lad, and symptoms of hectic, excision was decided upon, and performed on May 21st.

*Operation.*—A long, straight incision was made from above, downwards, over the great trochanter. After a considerable thickness of soft and infiltrated tissue had been cut through, the upper portion of the femur was exposed. The head of the bone was found to be firmly fixed in the cotyloid cavity. By forcibly adducting and flexing the limb, the end of the femur was thrust through the wound. The head was denuded of cartilage, flattened, and irregular in shape; the neck and great trochanter were also much diseased. The saw was applied, at first, just below the great trochanter; but as the cancellated tissue was found diseased at the level of this section, another slice of the femur was removed. The acetabulum was quite bare of cartilage, but the form and depth of the cavity were but slightly altered. A gouge was applied to the exposed bone, forming the floor and margins of the cavity; but, as this was firm and vascular, only a small portion was removed. The edges of the incision were brought together by sutures, and the limb fixed by the usual long splint.

For the first three weeks after the operation the patient did well. The emaciation persisted, but the hectic ceased; the appetite returned, and the general condition was much improved. The wound, though extensive, became rapidly filled up by granulations. A great tendency to shortening and adduction was counteracted by abducting the limb and keeping it extended by weights.

At the end of June, there was an unfavorable change. The upper part of the right thigh began to swell and to become œdematous, and there was an increase in the amount of discharge. His rest and mental condition were much disturbed by severe pains, which radiated from the seat of operation, along the front of the thigh and leg to the foot. These were relieved only by repeated injections of morphia under the skin. From the 24th of June the patient became gradually worse. Extreme general emaciation was marked in the lower limbs, especially in the left leg and foot, by extensive anasarca. The purulent discharge from the right thigh persisted, and the œdema and severe pain over the iliac region indicated deep-seated suppuration in the pelvis. At the end of August, the patient passed suddenly into a state of unconsciousness, from which he but partially recovered; and, after occasional delirium, he died on the evening of September 5th.

*Post Mortem Examination.*—Lower limbs and scrotum very anasarcous. In front of right femur two large abscesses leading up to the seat of operation; back part of thigh traversed from gluteal region to popliteal space by a third purulent sac. Upper extremity of right femur closely embraced by fibrous and muscular tissues; the surface left by the saw covered a thick layer of tough, organized lymph. Acetabulum occupied by upper part of femur; osseous tissue around the cavity much diseased; and the floor perforated by three orifices, two of which communicated with intra-pelvic abscesses, the third with a large collection of pus in the right iliac fossa. The portions of innominate bone, beyond the carious and blackened tissue, surrounding the cotyloid cavity, are quite sound. Both lungs occupied by very numerous nodules of miliary tubercle. Right kidney lardaceous; the left affected with extensive scrofulous disease. Liver pale; weight 2¾ lbs.—*Lancet*, Oct. 9th, 1869.

---

Death by Hemorrhage from Cerebral Tumors (under the care of Dr. Hughlings Jackson).—The modes of death from cerebral tumors are very various. One patient suffers from the tumor slowly, one nervous symptom following another; another dies of what is called brain fever; a third is suddenly killed by hemorrhage from the tumor. Again, some patients die without any symptoms referable to the head, and a tumor is found *post mortem*. Death by hemorrhage deserves careful recognition. If we have a good history, these cases give us little trouble in diagnosis.

A boy, aged nine, was under Dr. Hughlings Jackson's care, at the London Hospital, for paralysis of the third nerve, on one side, and hemiplegia on the other. These symptoms had come on slowly, with headache; and there was a double optic neuritis. He died during the night of effusion of blood, from a tumor of the crus cerebri, which caused the paralytic symptoms. The palsy of the third nerve on one side, and of the arm and leg on the other, pointed to disease of the crus cerebri. The gradual onset of the paralysis, and its complication with double optic neuritis, made it certain that the disease was of some *coarse* kind. The age of the patient rendered it most probable that this coarse disease was tumor. His sudden death led to the inference of hemorrhage from tumor.

The following case might easily have given trouble in diagnosis, had not the patient's previous history been known. A man, 23 years of age, under the care of Dr. Ramskill, had convulsive attacks; he suffered intense pain in his head, and there was double optic neuritis. It was almost certain, from these symptoms, that there was intracranial tumor. He was doing very well, being about the ward. He had a good appetite, and was able to read, when one night he was seized with a convulsion, became very deeply comatose, and died in two or three hours. Dr. Sutton found at the autopsy a gliomatous tumor of the fore part of the left anterior cerebral lobe, with recent effusion of blood to the extent of several ounces.

Had this man been brought to the hospital under like circumstances to those of the patient whose case is next related, the diagnosis would have been impossible, unless, perhaps, the ophthalmoscope had been used. If we find optic neuritis in a young, healthily-built patient, who has become comatose after a convulsion, we should, Dr. Hughlings Jackson thinks, be warranted in diagnosing tumor, but we could not say there was hemorrhage from that tumor. It is well-known that patients, the subjects of organic disease of many kinds, probably of any kind affecting one cerebral hemisphere, are liable to coma, after attacks of simpler convulsions, *i.e.*, convulsive attacks, without any effusion of blood.

But if the patient with optic neuritis were *very deeply* comatose, especially if he were so after but one sudden convulsion; if the fit was not known to have begun by a deliberate "aura," in the face, arm, or leg, and especially if the patient had had fits so beginning before; if there were no discoverable hemiplegia, we should fear hemorrhage from a tumor, and we should believe the patient would die. But we have not always such helps. The diagnosis is not always ready made. In the following case, the patient was not young, there was no known convulsion, and there was no changes in the optic disks, and the history Mr. Leach supplied was not obtained until after the autopsy. It is needless to say that the diagnosis of hemorrhage, from cerebral tumor, was, in this case, impossible. It has no less value on that account.

A man, 56 years of age, was discharged on July 1st, from the "Dreadnaught," for a simple infraction of the rules. The only symptom the man then had was pain in the head. He was brought, on July 10th, in a comatose state, to the London hospital. He died there that night, at 1.30 P.M. All the police knew was that the poor fellow had been picked up in the street the day before, and taken to his lodgings, when he was supposed to be tipsy. As he did not get up next morning, he was looked after at midday. At the autopsy, a vascular tumor was found in the posterior lobe of the left hemisphere, close upon the middle cornu, and in the descending cornu. In the lateral ventricle was an effusion of blood to the extent of several ounces, which had presumably come from the tumor, as no part of the brain was broken up, except the wall of the middle cornu, near the tumor.—*The Lancet*, Oct. 23, 1869.—*Medical News.*

